# A GC–MS-Based Urinary Metabolomic Profiling to Identify Potential Biomarkers of Metabolic Syndrome

**DOI:** 10.1021/acsomega.5c08140

**Published:** 2025-10-17

**Authors:** Juhan Pak, Mee-Hyun Lee, Seong-Eun Park, Soobin Bae, Gayoun Lee, Yanghee You, Gi Dae Kim, Chang-Su Na, Hong-Seok Son

**Affiliations:** † Department of Biotechnology, 34973 Korea University, Seongbuk-gu, Seoul 02841, South Korea; ‡ 34944 College of Korean Medicine, Dongshin University, Naju, Jeollanam-do 58245, South Korea; § Department of Food and Nutrition, Kyungnam University, Changwon, Gyeongsangnam-do 51767, South Korea

## Abstract

Metabolic syndrome (MetS) is a multifactorial condition characterized by central obesity, dyslipidemia, hypertension, and insulin resistance, increasing the risk of cardiovascular disease and type 2 diabetes. Despite its clinical significance, current diagnostic methods rely on invasive blood-based assessments. This study investigates the potential of urinary metabolomics as a noninvasive alternative for MetS diagnosis. Using gas chromatography–mass spectrometry (GC–MS), we analyzed urinary metabolites from 127 individuals classified into Normal, Borderline (BL), and MetS groups based on clinical diagnostic criteria. A total of 80 metabolites were identified, and partial least-squares discriminant analysis (PLS–DA) revealed distinct metabolic profiles between groups. Key metabolites, including glucuronate, galacturonic acid, and cystine, showed significant associations with MetS and its diagnostic components. Pathway analysis indicated metabolic perturbations primarily in carbohydrate, amino acids, and fatty acid metabolism. Furthermore, receiver operating characteristic (ROC) curve analysis demonstrated that a selected panel of urinary metabolites improved MetS classification accuracy. These findings suggest that urinary metabolomics profiling can provide novel biomarkers for MetS, offering a promising approach for noninvasive screening and early detection.

## Graphical abstract

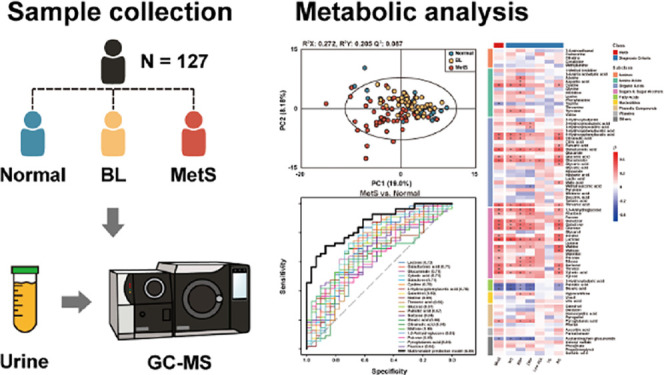

## 1 Introduction

Metabolic syndrome (MetS) is a complex metabolic disorder characterized by the concurrent presence of multiple risk factors including abdominal obesity, elevated blood pressure, high fasting blood glucose levels, elevated triglycerides, and low levels of high-density lipoprotein (HDL) cholesterol.^1^ Globally, MetS has become a major public health challenge with an estimated prevalence ranging from 10 to 84% depending on the ethnicity, age, and sex/gender.^2,3^ Individuals with MetS are at a significantly higher risk of developing cardiovascular disease and type 2 diabetes, highlighting the need for early diagnosis and effective management strategies.^4^

Traditional diagnostic approaches for MetS primarily rely on clinical assessments of established risk factors requiring blood samples to measure triglycerides, HDL cholesterol, and fasting glucose levels.^5^ However, blood sampling is invasive and may cause discomfort, making it less suitable for frequent monitoring or large-scale screening. Additionally, blood specimens in clinical laboratories can be rejected due to issues such as clotting and hemolysis,^6^ which may affect test accuracy and require repeat sampling. Given these limitations, urinary metabolomics has emerged as a promising alternative for gaining mechanistic insights into MetS. As a noninvasive and easily accessible biofluid, urine enables large-volume collection with high participant compliance and serves as a rich metabolic reservoir, reflecting systemic metabolic fluctuations and accumulating metabolic byproducts from multiple pathways. Additionally, urine generally contains higher metabolite concentrations than plasma, further enhancing its potential for biomarker discovery.^7^

Recent studies have widely employed metabolic profiling techniques such as nuclear magnetic resonance (NMR) and liquid chromatography–mass spectrometry (LC–MS) to characterize urinary metabolic signatures of MetS.^8–10^ These approaches have provided valuable insights into metabolic alterations linked to MetS and have demonstrated the potential of urinary metabolomics for disease classification. However, most studies have focused on distinguishing MetS from non-MetS groups, rather than elucidating how urinary metabolites relate to individual MetS diagnostic criteria. Moreover, the five diagnostic components do not contribute equally to MetS pathophysiology, as central obesity and insulin resistance are considered primary drivers of metabolic dysfunction, whereas dyslipidemia and hypertension may develop as secondary manifestations.^11^ Despite what is already known, how urinary metabolites are differentially linked to each MetS diagnostic criterion remains unclear. Therefore, a more detailed investigation of the metabolic disturbances associated with each MetS component is necessary to better understand its pathophysiology and improve diagnostic accuracy.

To achieve this, we employed an untargeted urinary metabolomics approach to systematically analyze urinary metabolite profiles and their associations with individual MetS diagnostic criteria. Gas chromatography–mass spectrometry (GC–MS) with derivatization is particularly well-suited for detecting a broad range of polar metabolites, including sugar derivatives, organic acids, and amino acids.^12^ Since urine is naturally enriched with these metabolites, GC–MS serves as a robust tool for detecting metabolites associated with MetS. Furthermore, to identify key metabolic signatures and their relationships with MetS components, we applied multivariate analysis and correlation analysis to determine metabolites associated with specific diagnostic criteria. Through this approach, this study enhances our understanding of MetS pathophysiology and contributes to the identification of potential metabolic biomarkers for improved diagnosis and risk assessment.

## 2 Material and Methods

### 2.1 Sample Cohorts and Collection

This study was approved by the Ethics Committee of Naju Korean Medicine Hospital of Dongshin University (NJ-IRB-013). A total of 127 participants were recruited from volunteers at the Naju Korean Medicine Hospital, Dongshin University. Urine samples were collected fresh, midstream, in the morning after an overnight fast using sterile 500 mL beakers from each participant. Urine was transferred into Eppendorf tubes and immediately stored at −80 °C until analysis. In addition to urine samples, detailed health examination was conducted, including the five clinical criteria used to diagnose MetS: waist circumference (WC), blood pressure (systolic and diastolic; SBP, DBP), HDL cholesterol, triglyceride (TG) levels, and fasting blood glucose (BG). Based on these data, participants were categorized into three groups: the normal group (Normal), with no risk factors; the Borderline group (BL), with one or two abnormal values; and the MetS group (MetS), with three or more abnormal values. The diagnostic criteria used were based on the National Cholesterol Education Program Adult Treatment Panel (NCEP ATP III)^13^ with the abdominal obesity cutoff adjusted to better fit the Korean population: WC ≥ 90 cm for men or ≥85 cm for women; SBP ≥ 130 mmHg and/or DBP ≥ 85 mmHg or use of antihypertensive medication; HDL cholesterol <40 mg/dL for men or <50 mg/dL for women or use of HDL-raising medication; TG ≥ 150 mg/dL or use of triglyceride-lowering medication; and BG ≥ 100 mg/dL or use of glucose-lowering medication. Information on the use of antihypertensive, lipid-lowering, and antidiabetic medications was collected. Almost all individuals taking these medications were classified into the MetS or borderline groups, consistent with the diagnostic definition. Only one participant in the normal group reported lipid-lowering therapy; however, this individual did not meet the threshold of three criteria and was therefore appropriately classified as normal.

### 2.2 Sample Preprocessing

The metabolic analysis protocol and GC–MS conditions were similar to those described in a metabolomic study^14^ with slight modifications. The urine samples were carefully thawed in an ice bath before analysis. Urine metabolites were extracted by adding 900 μL of methanol to 100 μL of urine samples. The samples were vortexed for 5 min, followed by centrifugation at 15,928 *g* for 10 min at 4 °C. After the centrifugation, 400 μL of supernatant was collected, and 30 μL of each sample were pooled for quality control (QC) samples. To the collected supernatant samples, 20 μL of ribitol (0.5 mg/mL) was added as the internal standard, then the samples were subjected to centrifugal vacuum concentration for 12 h.

### 2.3 Sample Derivatization

The dried samples were derivatized by adding 100 μL of methoxyamine hydrochloride solution (20 mg/mL in pyridine). The samples were subjected to ultrasonication for 20 min in an ice bath using a Powersonic 520 (Hwashin, Seoul, Republic of Korea) to ensure complete dissolution. Following sonication, each mixture was vortexed for 1 min and 30 s, then incubated with shaking at 75 rpm for 90 min at 30 °C. After the first incubation, 50 μL of *N*-methyl-*N*-trimethylsilyl-trifluoroacetamide (MSTFA) was added to each mixture. The samples were briefly vortexed, followed by a second incubation with shaking at 75 rpm for 30 min at 37 °C. The mixtures were then centrifuged at 13,572 *g* for 5 min at 4 °C to separate any precipitates. A 1.5 mL vial with a 100 μL insert was prepared, into which 80 μL of the supernatant was transferred for GC–MS analysis.

### 2.4 GC–MS Analysis

The derivatized samples were analyzed on a GC–MS system (QP2020, Shimadzu, Kyoto, Japan), utilizing an Rtx-5MS fused silica capillary column (30 m × 0.25 mm ID, J&W Scientific, Folsom, CA, USA) for metabolite separation. The front inlet temperature was set at 230 °C. The column temperature program began with an isothermal hold at 80 °C for 2 min, followed by a ramp of 15 °C/min up to 330 °C, where it was held isothermally for 6 min. The transfer line and ion source temperatures were maintained at 250 and 200 °C, respectively. Ionization was carried out using a 70 eV electron beam. Helium served as the carrier gas with a flow rate of 1 mL/min. Data acquisition was set at 20 scans per second, covering a mass range of 85–500 *m*/*z*. To evaluate system performance and stability, as well as the reproducibility of the sample preparation process QC samples were analyzed at intervals of every 20 samples during the run. Chromatograms and mass spectra were generated using Shimadzu GC Solution software (Shimadzu, Kyoto, Japan).

### 2.5 Data Processing

GC–MS raw data were converted to ABF format. MS-DIAL ver. 4.9.221218, together with an open-source publicly available electron ionization (EI) spectra library, was used for raw peak extraction, baseline filtering, baseline calibration, peak alignment, deconvolution, peak identification, and peak height integration, following established methods.^15^ Peak detection involved an average peak width of 20 scans and a minimum peak height of 1,000 amplitudes. A sigma window value of 0.5 and an EI spectra cutoff of ten amplitudes were used for deconvolution. For metabolite identification, retention time tolerance was set at 0.5 min, *m*/*z* tolerance at 0.5 Da, EI similarity cutoff at 90%, and identification score cutoff at 90%. During alignment, the retention time tolerance was set to 0.075 min and the retention time factor to 0.5. Kováts retention index (RI) values were determined from an *n*-alkane series (C7–C40) analyzed under the same GC–MS conditions and compared to experimental RIs. To increase confidence, several metabolites were directly validated using authentic standards analyzed in-house, with retention times and fragmentation patterns matched against our in-house spectral library. These metabolites are indicated in Supplementary Table S2 under the column “Standard validation (O).” In addition, the mass spectra of identified metabolites were compared to those of standard reagents and annotated using publicly available spectral libraries, including NIST ver. 20.0 and the Human Metabolome Database (https://www.hmdb.ca; accessed on 6 Aug 2024). Collectively, these approaches ensured high-confidence metabolite identification.

To account for potential variations in sample intensities, the metabolite intensities were normalized as a ratio to the total sum of all metabolite intensities within each sample. Multivariate statistical analysis of the final GC–MS data was performed using partial least-squares discriminant analysis (PLS–DA) to visualize metabolite variance. The analysis was conducted with SIMCA-P 17.0 software (Umetrics, Umea, Sweden). The quality of the model was assessed using *R* ^2^ *X* (variance explained in predictor variables), *R* ^2^ *Y* (variance explained in response variables), and *Q* ^2^ (predictive accuracy of the model) values.

### 2.6 Pathway Analysis

For the metabolites that were significantly different between the Normal and MetS groups, a quantitative enrichment analysis (QEA) and pathway topology analysis were performed. Enrichment and pathway analyses using the KEGG pathway database were conducted with MetaboAnalyst 6.0, a web-based platform (https://www.metaboanalyst.ca; accessed on 31 Oct 2024).

### 2.7 Statistical Analysis

Statistical analyses were performed using GraphPad Prism software package, version 9.4.1 (GraphPad Software Inc., San Diego, California, USA). The normality of the data set was assessed using Shapiro-Wilk test. For parametric and nonparametric data analysis, the Normal, BL, and MetS groups were compared by a one-way analysis of variance (ANOVA), Brown–Forsythe test, and Kruskal–Wallis test, respectively. Multiple comparisons were performed using the two-stage step-up method of Benjamini, Krieger, and Yekutieli, or Dunn’s tests. The correlation analysis and heatmap generation were performed using R Statistical Software, version 4.4.1 (R Foundation for Statistical Computing, Vienna, Austria). Spearman’s rank correlation coefficient (ρ) was employed to investigate the association between metabolite intensities and MetS diagnostic criteria. Correlations were color-coded in the heatmap (red for positive, blue for negative correlations), with significant associations (*p* < 0.05) marked by an asterisk (*). To assess the diagnostic accuracy of identified metabolomic features, receiver operating characteristic (ROC) curves were constructed using the roc function in the pROC package^16^ based on logistic regression models. Diagnostic performance of models was quantified by calculating the area under the curve (AUC). All analytical results were visualized using ggplot2 package.^17^

## 3 Results

### 3.1 Participant Classification and General Characteristics

A total of 127 participants were included in the study, categorized into three groups: Normal (*n* = 31), BL (*n* = 41), and MetS (*n* = 55) based on the diagnostic criteria for MetS. The participants’ general characteristics, including WC, BP (systolic and diastolic), HDL cholesterol, TG, and BG, were compared across the three groups (Table 1). The MetS group exhibited significantly higher WC, BP, TG, and BG levels, while HDL cholesterol levels were lower compared to the Normal and BL groups (*p* < 0.05 for all comparisons). Given that the MetS group had a significantly higher age than the other groups, we evaluated whether age itself influenced urinary metabolite levels. Linear regression and Spearman correlation analyses were performed to assess the associations between age and identified metabolites (Table S1). However, all metabolites exhibited weak association with age (ρ < 0.3), suggesting that age-related metabolic variations alone do not fully explain the metabolic differences observed in MetS individuals.

**Table 1:** Clinical Characteristics of the Normal, Borderline (BL), and Metabolic Syndrome (MetS) Groups^a^

group	**Normal**(*n* = 31)	**BL**(*n* = 41)	**MetS**(*n* = 55)
age	38.3^a^ ± 14.7	47.8^b^ ± 12.1	54.3^b^ ± 11.6
sex			
male, *n* (%)	16 (51.6%)	22 (53.7%)	26 (47.3%)
female, *n* (%)	15 (48.4%)	19 (46.3%)	29 (52.7%)
BMI, kg/m^2^	22.2 ± 2.7	23.7 ± 2.6	29 ± 3.5
WC, cm	73.8^a^ ± 7.0	78.8^a^ ± 11.9	92.8^b^ ± 7.6
SBP, mmHg	114.9^a^ ± 9.2	125.2^b^ ± 16.3	137.3^c^ ± 12.6
DBP, mmHg	69.5^a^ ± 6.8	75.9^b^ ± 11.6	83.7^b^ ± 12.3
HDL, mg/dL	66.5^a^ ± 13.6	56.9^b^ ± 15.6	53.9^c^ ± 38.9
TG, mg/dL	78.2^a^ ± 25.2	104.9^a^ ± 62.8	203.5^b^ ± 126.1
BG, mg/dL	92.7^a^ ± 3.8	103.3^b^ ± 12.7	120.4^c^ ± 33.7

a Values are presented as mean ± standard deviation. Superscript letters indicate statistically significant differences between groups within the same row (*p* < 0.05). Groups sharing the same letter are not significantly different, while different letters indicate a statistically significant difference. BMI, body mass index; WC, waist circumference; SBP, systolic blood pressure; DBP, diastolic blood pressure; HDL, high-density lipoprotein; TG, triglyceride; BG, fasting blood glucose.

### 3.2 Key Metabolite Differences among Normal, BL, and MetS Groups

A total of 80 urinary metabolites were identified using untargeted GC–MS analysis (Table S2 **).** These metabolites spanned various metabolic classes, including amines, amino acids, organic acids, sugars and sugar alcohols, fatty acids, nucleotides, phenolic compounds and vitamins. The PLS–DA analysis using urinary metabolite data was performed to classify participants into three distinct groups: Normal, BL, and MetS. In the score plot, the MetS groups showed a clear separation from the Normal and BL groups, while the BL group largely overlapped with the Normal group, indicating limited distinction between these two groups based on urinary metabolite profiles (Figure 1A). This separation indicates that urinary metabolite profiles hold strong discriminative power for MetS. To assess the robustness of the PLS–DA model, permutation tests were performed. This result validates the predictive ability of the model (Figure 1B).

**Figure 1:**
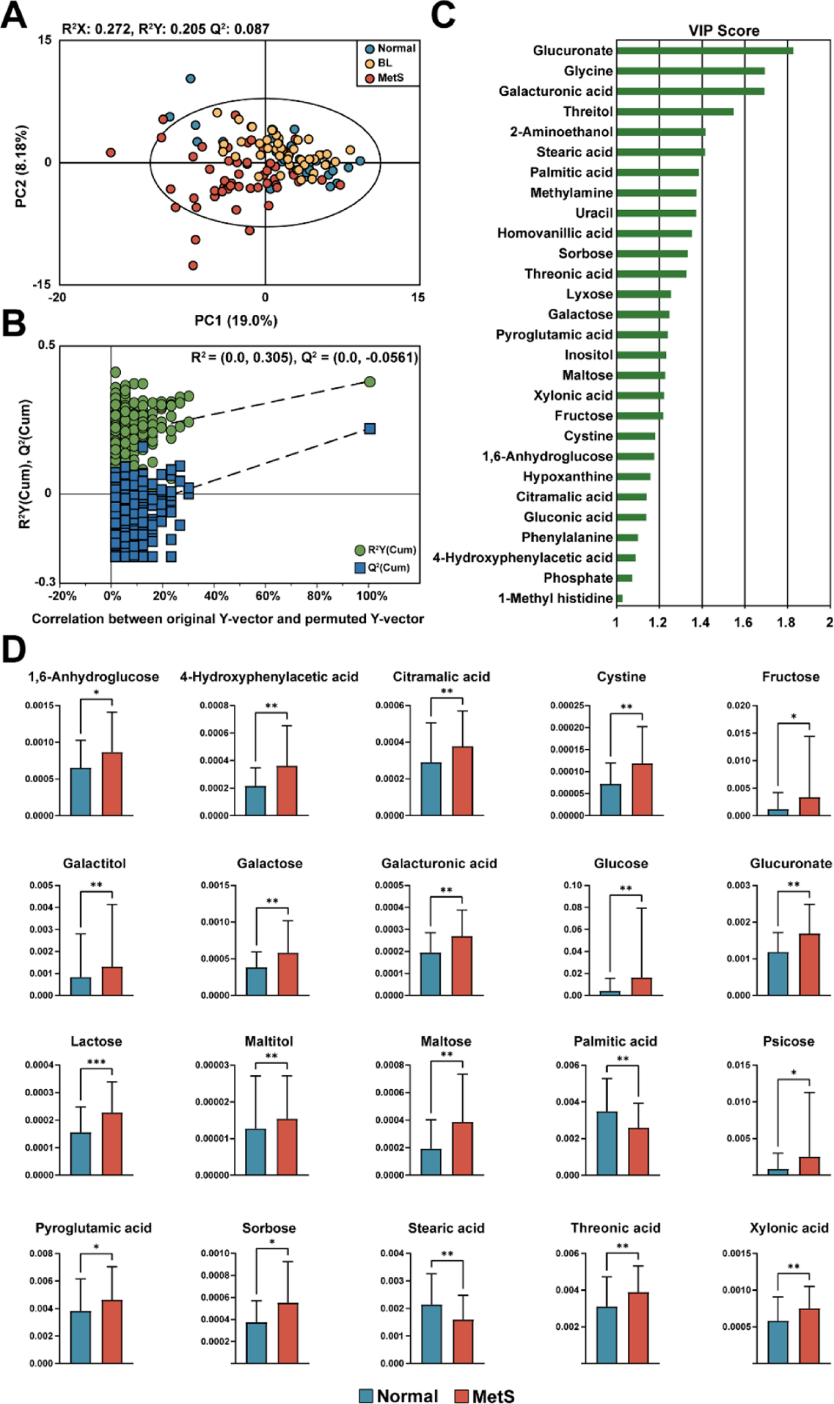
PLS–DA analysis and metabolite level differences among the groups. (A) PLS–DA score plot showing the clustering of participants into three groups: Normal (blue), BL (orange), and MetS (red) based on urinary metabolite profiles. Ellipses represent 95% confidence regions. (B) Permutation test plot validating the model’s predictive ability, with significantly higher *Q* ^2^ and *R* ^2^ values compared to permuted models, confirming the robustness of group separation. (C) Variable importance in projection (VIP) scores for metabolites contributing to group separation (VIP ≥ 1.0). (D) Bar plots of relative concentrations of metabolites significantly different among groups. Statistical significance was assessed by one-way ANOVA with FDR correction (*p < 0.05, **p < 0.01).

Metabolites with a variable importance in projection (VIP) score ≥ 1.0 were considered significant contributors to the group separation in the PLS–DA model. Among the 28 identified metabolites with VIP score above 1.0, glucuronate, glycine, and galacturonic acid displayed the highest VIP scores (Figure 1C). Further statistical analysis identified 20 metabolites that exhibited significant differences between the Normal and MetS groups, reinforcing their potential as metabolic markers (Figure 1D)**.** Urine samples from the MetS group had significantly higher levels of 1,6-anhydroglucose, 4-hydroxyphenylacetic acid, citramalic acid, cystine, fructose, galactitol, galactose, glucuronic acid, glucose, glucuronate, lactose, maltitol, maltose, psicose, pyroglutamic acid, sorbose, threonic acid, and xylonic acid, while lower levels of palmitic acid and stearic acid were observed. These findings indicate that specific urinary metabolites provide strong discriminatory power for identifying individuals with MetS.

### 3.3 Metabolic Alteration Associated with Abnormality in MetS Diagnostic Criteria

Multivariate analysis was conducted to illustrate metabolic differences and key contributing metabolites between normal and abnormal groups for each MetS diagnostic criterion (Figures S1–S5). To comprehensively illustrate the results of the multivariate analysis, 20 urinary metabolites that exhibited significant differences in MetS group were evaluated across diagnostic criteria (Figure 2). In Figure 2A, each diagnostic criterion column contains a contribution score, which was assigned based on whether the metabolite was significantly altered in the abnormal group. The MetS score represents the sum of the contribution scores across all five diagnostic criteria, indicating the net contribution of each metabolite to MetS. Among the metabolites that exhibited positive MetS scores, cystine, galacturonic acid, glucuronate, galactose, and xylonic acid were found to be significantly elevated in most of the diagnostic criteria suggesting their potential role as metabolic markers of MetS. Conversely, palmitic acid and stearic acid, which exhibited negative MetS scores, were higher in the normal group and had inverse associations with certain diagnostic criteria. To further illustrate the metabolic network underlying MetS, a network plot was constructed using metabolites significantly higher in MetS group and their associations with diagnostic criteria (Figure 2B). Among the diagnostic criteria, BP, WC, and BG exhibited the largest number of connections, suggesting that metabolic alterations associated with MetS are predominantly influenced by abnormalities in these criteria. In contrast, TG showed minimal associations with urinary metabolites in this study, indicating a weaker metabolic link compared to other diagnostic criteria. The heatmap (Figure 2A) also supports this observation, as TG contributed little to the overall metabolic score, with only one metabolite exhibiting significant association. This suggests that while TG is a key diagnostic component of MetS, its impact on urinary metabolomic shifts may be relatively limited compared to other factors such as glucose metabolism and blood pressure regulation.

**Figure 2:**
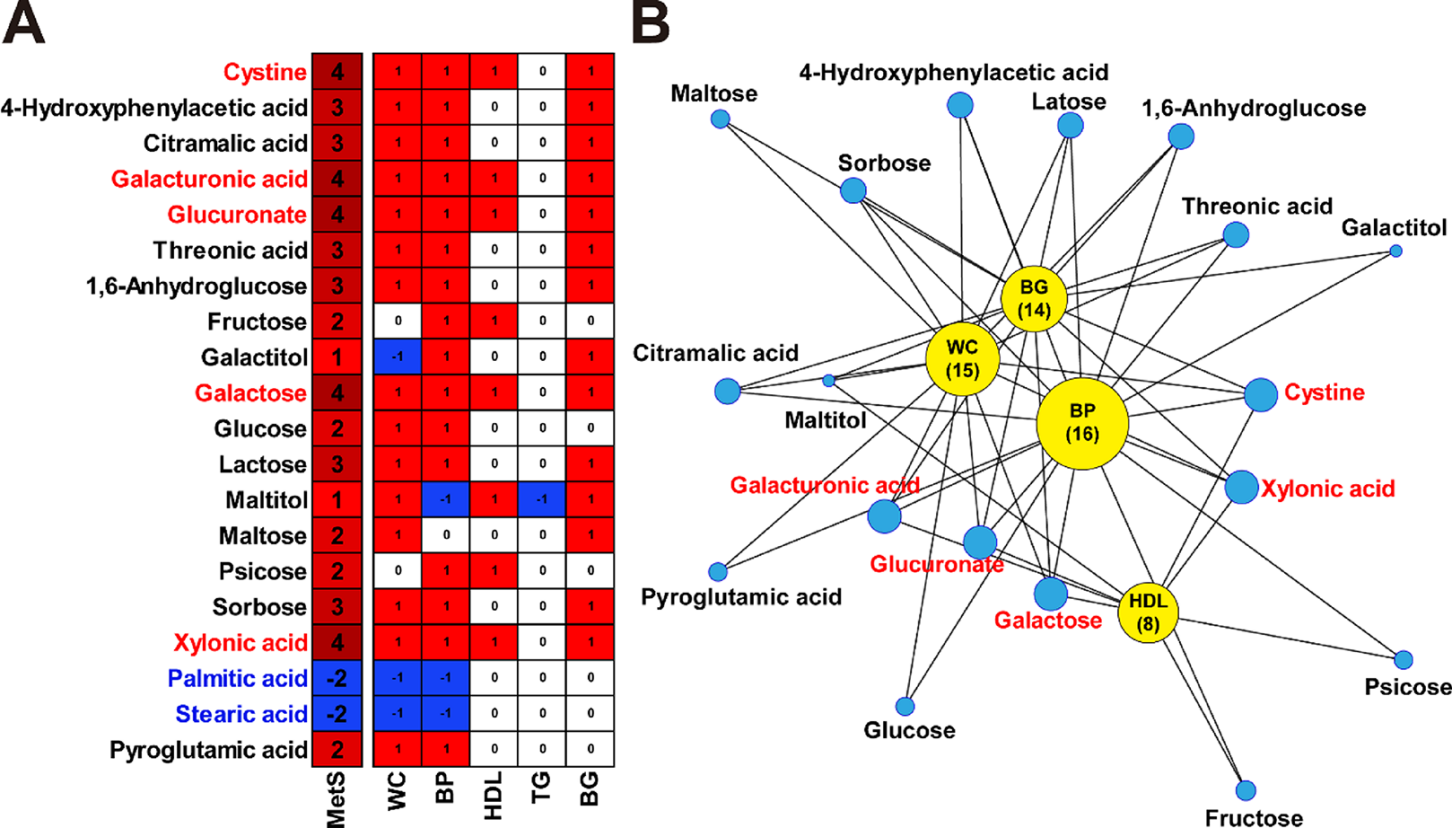
Contribution of urinary metabolites to MetS and its diagnostic criteria. (A) Heatmap representing the contribution scores of 20 urinary metabolites across the five diagnostic criteria of MetS: waist circumference (WC), blood pressure (BP), HDL cholesterol, triglycerides (TG), and blood glucose (BG). Contribution scores were assigned based on the metabolite’s significant level in the abnormal group for each criterion: +1 if significantly higher, −1 if significantly lower, and 0 if not significantly different. The MetS score (leftmost column) represents the sum of the contribution scores across all diagnostic criteria, reflecting the overall extent of metabolic alterations associated with MetS. (B) Network plot illustrating the associations between significant urinary metabolites and MetS diagnostic criteria. Yellow nodes represent diagnostic criteria, with their size proportional to the number of significantly associated metabolites in bracket. Blue nodes represent metabolites with node size scaled according to the MetS score, highlighting metabolites that exhibit stronger associations across multiple criteria.

### 3.4 Correlation between Metabolites and MetS Diagnostic Criteria

To explore the relationships between specific metabolites and the diagnostic criteria for MetS, a correlation analysis was conducted (Figure 3). The correlation heatmap provides an overview of associations between urinary metabolites, MetS, and its diagnostic criteria, including WC, BP, low HDL cholesterol, TG, and BG. The levels of several key metabolites, such as galacturonic acid, glucuronate, and lactose, showed strong positive correlations with MetS and all diagnostic criteria except for TG. In contrast, metabolites such as taurine, palmitic acid, stearic acid, and acetaminophen glucuronide exhibited strong negative correlations with MetS and its diagnostic criteria, suggesting these metabolites may be inversely associated with specific aspects of MetS. In the sugars and sugar alcohols group, most metabolites demonstrated significant positive correlations with MetS and its diagnostic criteria, particularly BP and BG. Notably, metabolites such as 1,6-anhydroglucose, galactitol, galactose, and glucose showed high positive correlations, underscoring the role of carbohydrate dysregulation in MetS. Additionally, several metabolites within the organic acid group also displayed significant correlations with MetS criteria. For instance, galacturonic acid and glucuronate showed significant positive correlations in most of the criteria. Among amino acids, alanine, aspartic acid, cystine, histidine, and tyrosine showed significant positive correlations with BP, whereas taurine demonstrated negative correlations across all criteria.

**Figure 3:**
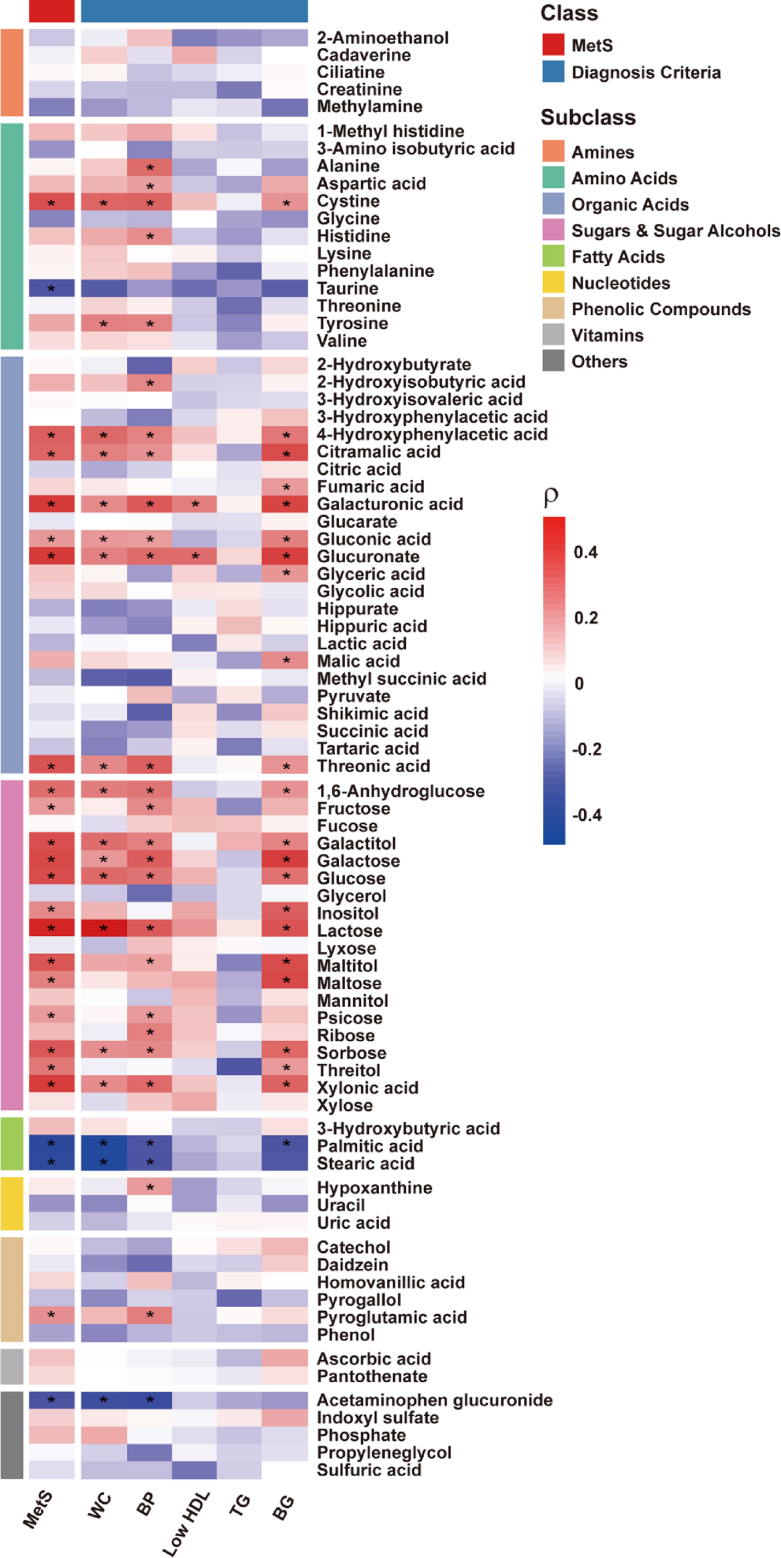
Correlation heatmap of urinary metabolites with MetS and its diagnostic criteria. The heatmap illustrates the Spearman correlation (ρ) between identified urinary metabolites and MetS as well as individual diagnostic criteria (WC, BP, HDL, TG, BG). Metabolites are categorized into subclasses, represented by the colored side bars. Positive correlations are shown in red, while negative correlations are in blue (*: *p* < 0.05).

### 3.5 Metabolic Pathway Associated with Metabolite Shifts in MetS

Metabolic pathway analysis using MetaboAnalyst revealed significant enrichment of pathways associated with MetS-related urinary metabolite shifts. QEA was performed on all detected urinary metabolites and their intensities to analyze their contribution to MetS-related metabolic shifts (Figure 4A and Table S3 **)**. Significant pathways identified by QEA (*p* < 0.05) included pentose and glucuronate interconversions, fatty acid biosynthesis, fatty acid degradation, fatty acid elongation, and biosynthesis of unsaturated fatty acids. These pathways, which involve both carbohydrate and lipid metabolism, highlight systemic metabolic disruption in MetS. Pathway topology analysis further emphasized the importance of pentose and glucuronate interconversions, fatty acid biosynthesis, and fatty acid elongation (*p* < 0.05), confirming their central role in MetS-related metabolic alterations (Figure 4B and Table S4 **)**. Additionally, pathways such as phenylalanine, tyrosine, and tryptophan biosynthesis, ascorbate and aldarate metabolism, galactose metabolism, and starch and sucrose metabolism had an impact score above 0.5, indicating their potential influence on metabolic dysregulation in MetS.

**Figure 4:**
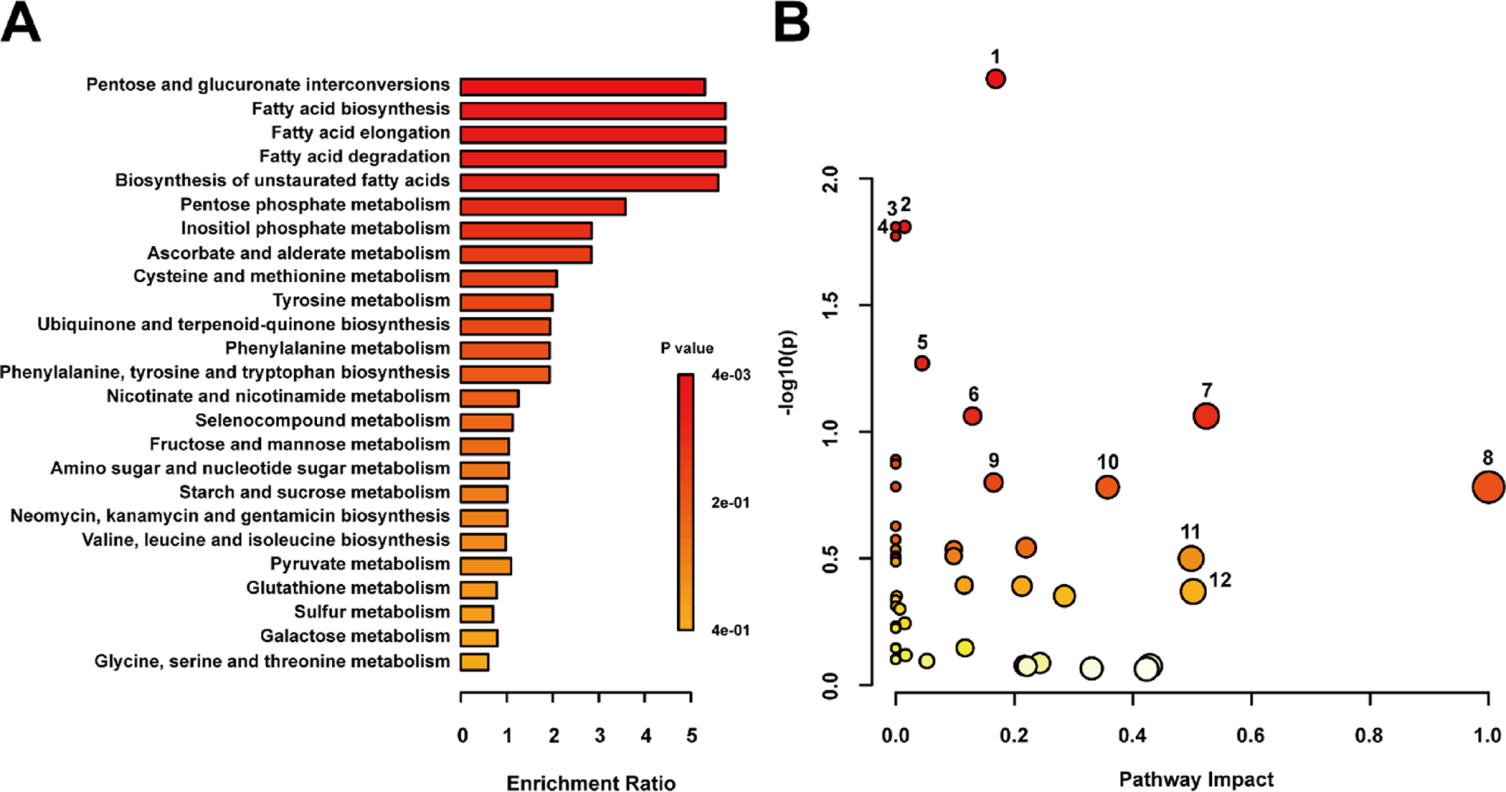
Graphical overview of quantitative enrichment analysis (QEA) and metabolic pathway topology analysis for metabolic pathways associated with MetS. (A) Bar chart of the QEA for the MetS urine metabotype against metabolic pathway-associated metabolite sets library in urine. Pathway associated metabolites sets are sorted based on enrichment ratio and *p* value. (B) Topological representation of metabolic pathways altered in MetS, including: (1) pentose and glucuronate interconversion, (2) fatty acid biosynthesis, (3) fatty acid elongation, (4) biosynthesis of unsaturated fatty acids, (5) pentose phosphate pathway, (6) inositol phosphate metabolism, (7) ascorbate and aldarate metabolism, (8) phenylalanine, tyrosine and tryptophan biosynthesis, (9) tyrosine metabolism, (10) phenylalanine metabolism, (11) starch and sucrose metabolism, (12) galactose metabolism. Graphs were generated by MetaboAnalyst 6.0 web-based software.

### 3.6 Comparative ROC Analyses of Metabolomic Markers for MetS Classification

To assess the discriminatory capacity of the key urinary metabolites in classifying MetS, ROC curve analyses were conducted. First, the diagnostic performance of selected urinary metabolites was assessed by comparing the MetS group to the non-MetS group (Normal and BL groups) (Figure 5A). Overall, the selected metabolites demonstrated limited discriminative power, with AUC values ranging from 0.59 to 0.73. However, certain metabolites positively correlated with MetS—lactose, glucose, galacturonic acid and glucuronate (Figure 3)—showed stronger diagnostic potential (AUC ≥ 0.70) compared to other metabolites. To assess the collective diagnostic capacity of these metabolites, a logistic regression model was developed, using the selected metabolites as predictors and MetS status as the dependent variable. The resulting ROC curve demonstrated an improvement in diagnostic accuracy compared to individual metabolites (AUC = 0.82). To further refine the model’s discriminatory capacity, an additional ROC analysis was conducted, this time excluding the BL group. The comparison between MetS and Normal groups revealed variations in predictive performance. Overall, diagnostic accuracy improved, with AUC values ranging from 0.64 to 0.73. Notably, lactose, galacturonic acid, glucuronate, xylonic acid, galactose, cystine, 4-hydroxyphenylacetic acid exhibited AUC values ≥ 0.70. The integrated multivariable prediction model further enhanced diagnostic accuracy compared to the previous model, yielding an AUC of 0.86. To further explore potential bias within the heterogeneous MetS group, we performed confusion matrix analyses stratified by individual diagnostic criteria (Table S5). The results showed heterogeneity in model performance, with higher sensitivity observed for WC and BG abnormalities, while specificity was lower for HDL- and TG-related abnormalities. These findings suggest that urinary metabolomic profiles may better capture adiposity- and glucose-related disturbances than lipid-related abnormalities. Importantly, permutation tests (1,000 iterations) confirmed that the observed AUROC values for both comparisons (MetS vs Normal & BL and MetS vs Normal) were significantly higher than expected under the null distribution (*p* < 0.001), supporting the robustness of our classification models (Figure S6).

**Figure 5:**
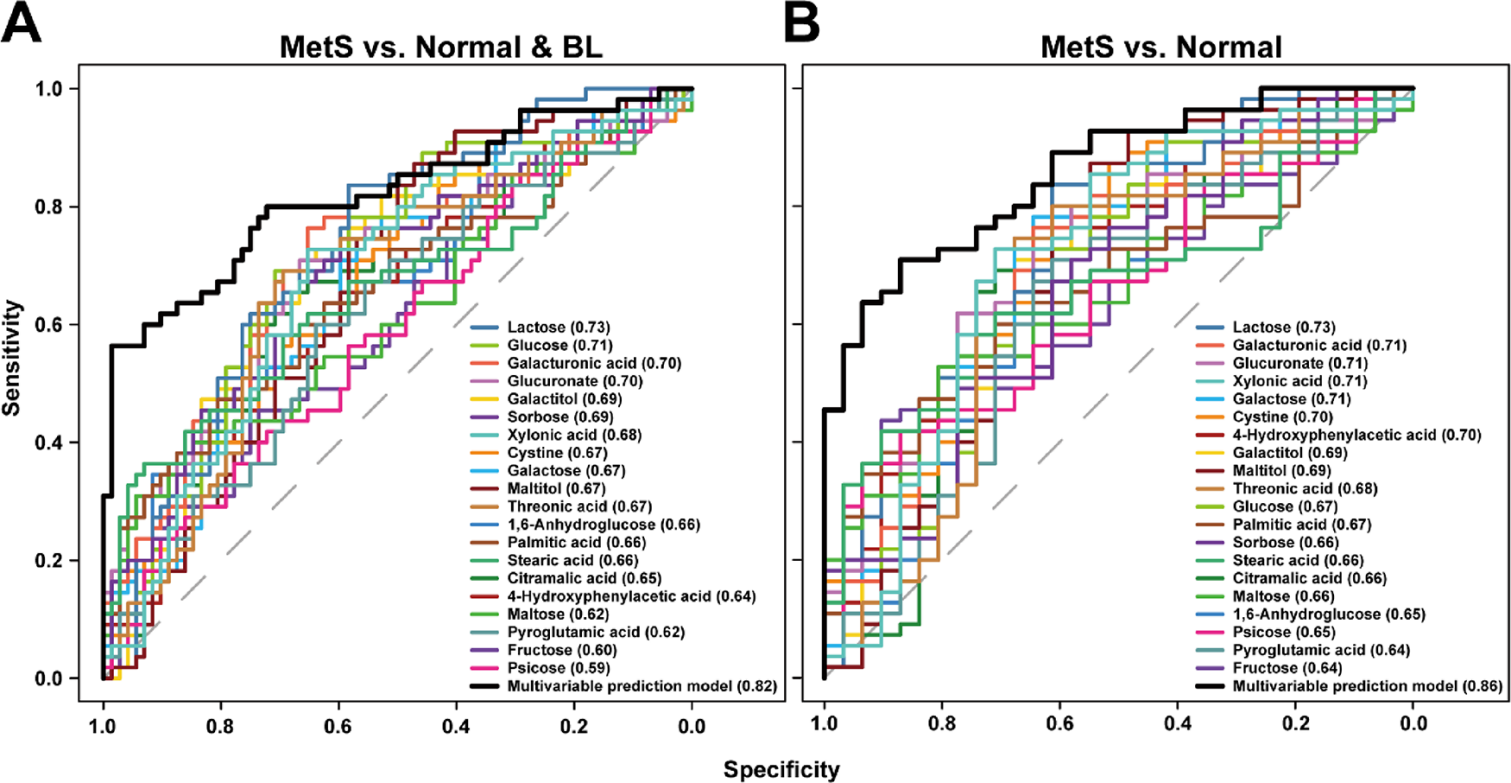
Receiver operating characteristic (ROC) curve analyses of the identified urine metabolome for MetS prediction. (A) ROC curves distinguishing the MetS group from Normal + BL groups. (B) ROC curves distinguishing MetS group from Normal group alone. Each curve represents the diagnostic performance of individual biomarkers or multivariable prediction models. The area under the curve (AUC) values reflects the ability of the urine metabolome to discriminate MetS from other groups. The AUC values of the ROC curves for each metabolite are indicated in parentheses following the respective metabolite names. The dashed line indicates the random classification boundary (AUC = 0.5).

## 4 Discussion

This study identified distinct urinary metabolic alterations associated with MetS using an untargeted GC–MS approach leading to the identification of 80 metabolites. Among these, 20 metabolites exhibited significant differences in the MetS group. Further analysis of these metabolites in the abnormal groups of each diagnostic criterion revealed that the five diagnostic criteria did not contribute equally to MetS. BP, WC, and BG exhibited the strongest associations with urinary metabolites, highlighting their central roles in MetS-related metabolic dysfunction. In contrast, HDL cholesterol and TG showed weaker correlations with urinary metabolites. These results suggest that urinary metabolite alterations primarily reflect metabolic disruptions related to hyperglycemia, and obesity, rather than lipid imbalances. Although the MetS group had a significantly higher age than the other groups, our correlation analysis demonstrated that age-related metabolic variations were minimal, indicating that the observed metabolic shifts are more likely driven by disease pathology rather than aging. Metabolite shifts were particularly evident in carbohydrate, amino acid, and fatty acid metabolism, indicating systemic metabolic dysregulation in MetS. The pathway analysis further supports this notion by highlighting mechanistic links between altered pathways and MetS progression. For example, perturbations in pentose and glucuronate interconversions reflect dysregulated carbohydrate metabolism and impaired glucuronidation capacity, which may exacerbate hyperglycemia and oxidative stress.^18^ Alterations in fatty acid biosynthesis and elongation pathways are closely tied to lipid accumulation and insulin resistance, central mechanisms of MetS, as evidenced by increased fatty acid synthesis contributing to triglyceride overload and metabolic dysfunction.^19^ Moreover, amino acid–related pathways, including phenylalanine, tyrosine, and tryptophan metabolism, have been associated with inflammation, oxidative stress, and vascular dysfunction in metabolic disorders.^20–22^ Together, these pathway alterations not only reflect the metabolic complexity of MetS but also point to biochemical processes that may serve as early biomarkers and intervention points for prevention or treatment strategies. Interestingly, the metabolite profiles of the BL group largely overlapped with those of the Normal group, suggesting that metabolic alterations in BL individuals may be subtle and not yet fully distinguishable. This overlap may reflect the transitional nature of the BL state, in which clinical risk factors are present but systemic metabolic dysfunction has not yet become pronounced. Although this reduces discriminatory power between BL and Normal groups, it underscores the clinical utility of metabolomics in detecting early metabolic disturbances before overt MetS development. Such insights may support the use of urinary metabolomics as a noninvasive tool for risk stratification and for monitoring individuals who may benefit from early preventive interventions.

Metabolites from the sugar and sugar alcohol group—particularly fructose, galactose, glucose, and lactose—showed a strong positive correlation with WC, BP, and BG. These sugars are central to energy metabolism and have well-established links to obesity, hyperglycemia, and, to a lesser extent, hypertension. Elevated glucose and fructose levels are commonly observed in individuals with increased adiposity and impaired glucose regulation, as these sugars promote energy storage and lipogenesis.^23,24^ Fructose, in particular, contributes to visceral fat accumulation and hepatic lipid synthesis, exacerbating insulin resistance.^25^ Similarly, elevated galactose levels may reflect impaired carbohydrate metabolism and increased reliance on alternative sugar pathways due to insulin resistance.^26^ Galactose metabolism has also been linked to oxidative stress and inflammation, both of which are key factors in obesity and hyperglycemia. These findings reinforce the role of excessive sugar metabolism in metabolic dysregulation and suggest that urinary sugars could serve as early biomarkers of MetS.^27^ Significant correlations were also identified between cystine, tyrosine, and taurine levels and MetS diagnostic criteria. Specifically, cystine and tyrosine exhibited positive correlations with WC and BP, while taurine showed consistent negative correlations across all criteria. Cystine, a key precursor of glutathione, plays a crucial role in oxidative stress regulation.^28^ Elevated cystine levels may indicate increased oxidative stress, a hallmark of obesity and MetS, which promotes systemic inflammation and metabolic dysregulation.^21^ Tyrosine, another amino acid frequently associated with obesity and insulin resistance, is involved in catecholamine synthesis and metabolic stress response.^29,30^ Elevated tyrosine levels have been linked to metabolic stress and impaired fat metabolism. In contrast, taurine levels were significantly lower in the MetS group, highlighting its potential protective role. Taurine has been recognized for its antioxidative and anti-inflammatory properties, particularly in supporting vascular health and reducing hypertension risk.^31^ Together, these findings suggest that specific amino acids may play dual roles in MetS, either exacerbating or mitigating metabolic dysfunction, making them potential therapeutic targets. We observed a significant reduction in urinary palmitic acid and stearic acid levels in the MetS group. While palmitic acid is a major product of de novo lipogenesis and is typically elevated in MetS, its lower urinary levels suggest a metabolic shift rather than systemic depletion.^32^ A possible explanation is increased conversion of palmitic acid and stearic acid via stearoyl-CoA desaturase-1, leading to higher production of monounsaturated fatty acids such as palmitoleic acid and oleic acid.^33^ Additionally, altered lipid metabolism in MetS may result in increased storage of these fatty acids in adipose and hepatic tissues, reducing their urinary excretion.^34–36^ Moreover, several studies have reported impaired or incomplete fatty acid β-oxidation in insulin-resistant states, which may further contribute to altered fatty acid turnover and reduced urinary appearance.^37,38^ These findings suggest that reduced urinary fatty acid levels in MetS reflect metabolic adaptations, including enhanced lipid storage, fatty acid desaturation, and altered β-oxidation, rather than a simple depletion of systemic fatty acids. Further lipidomic studies are needed to clarify these metabolic shifts and their implications for MetS progression.

The diagnostic potential of urinary metabolomics for MetS was also evaluated. In our study, individual urinary metabolites exhibited moderate diagnostic accuracy (AUC = 0.59–0.73), whereas combining multiple metabolites in a logistic regression model substantially improved prediction accuracy (AUC = 0.82–0.86). Notably, a previous study using UPLC-Q-TOF/MS-based plasma lipidomics identified three phosphatidylcholine species as MetS biomarkers, each exhibiting satisfactory diagnostic values (AUC > 0.7).^39^ Similarly, a plasma metabolomics study utilizing machine learning-based ROC analysis reported an AUC of 0.75 for distinguishing cardiovascular risk in MetS patients.^40^ These findings, along with our results, suggest that a biomarker panel approach could enhance diagnostic sensitivity and specificity, providing a promising noninvasive alternative to conventional blood-based biomarkers for routine screening and early detection. Given the ease of urine collection and the ability to capture metabolic changes over time, urinary metabolomics holds significant potential for large-scale population screening and long-term metabolic health monitoring.

Despite these promising findings, this study has several limitations. The overall sample size (*n* = 127), while acceptable for an exploratory metabolomics investigation, is relatively small and may restrict the broader applicability of the findings. In addition, the distribution across groups was unequal, particularly the limited number of participants in the Normal group (*n* = 31), which may have reduced statistical power and introduced potential bias. Another limitation is the lack of detailed information on potential confounding factors such as diet, medication use, physical activity, and lifestyle habits, all of which can significantly affect urinary metabolite profiles. In particular, medication intake may directly or indirectly alter urinary metabolite profiles or introduce drug-related signals, which could confound interpretation of some metabolites identified as significant. This possibility should be carefully addressed in future studies by systematically incorporating medication records and, where possible, adjusting for their effects in metabolomic analyses. Future studies with larger and more balanced cohorts, along with comprehensive collection of lifestyle and clinical data, will be essential to validate and extend our results. In addition, integrating multiomics approaches, including lipidomics and proteomics, will provide a more comprehensive understanding of MetS pathophysiology. While LC–MS and NMR approaches have also been applied in urinary metabolomics, our use of GC–MS enabled high-confidence identification of sugars, amino acids, organic acids, and fatty acids, which are central to MetS-related metabolic alterations. Nevertheless, integrating GC–MS with other analytical platforms in future studies will broaden metabolome coverage and strengthen mechanistic insights. Taken together, our findings highlight the strong potential of urinary metabolomics as a diagnostic tool for MetS. Our predictive modeling demonstrated that combining multiple urinary metabolites significantly improved MetS classification accuracy, reinforcing its utility as a noninvasive approach. These findings collectively suggest that urinary metabolic profiling can provide valuable biochemical insights into MetS pathophysiology while offering a promising avenue for early detection and risk stratification.

## 5 Conclusions

This study identified significant metabolic alterations in MetS individuals, highlighting key disruptions in carbohydrate, amino acid, and fatty acid metabolism. By integrating GC–MS-based metabolomics with statistical modeling, we demonstrate the potential of urinary metabolites as noninvasive biomarkers for MetS. Urinary metabolites provide a dynamic metabolic shift, offering deeper insights into MetS-related metabolic dysfunction. These findings underscore the value of urinary metabolomic profiling not only in enhancing diagnostic accuracy but also in advancing our understanding of the metabolic mechanisms underlying MetS. Ultimately, this study reinforces the role of metabolomics in precision medicine and metabolic disease management, supporting the development of noninvasive diagnostic strategies for improved clinical application.

## Supplementary Material



## Data Availability

The data supporting the findings of this study are available from the corresponding authors upon reasonable request because of the large volume of raw data collected in this study.

## Notes

Correlation analysis, metabolite information, pathway enrichment analysis, confusion matrix-based performance metrics, multivariate analyses by diagnostic criteria, and permutation test validation of AUROC models (PDF)
