# Supplementary material for: A GC–MS-Based Urinary Metabolomic Profiling to Identify Potential Biomarkers of Metabolic Syndrome
Source: ACS Omega. 2025 Oct 17;10(42):50545–55. doi: 10.1021/acsomega.5c08140 (PMC12573147; doi:10.1021/acsomega.5c08140)
Supplement: Supplementary file 1 [file ao5c08140_si_001.pdf]

Supplementary Materials for:

A GC-MS-based urinary metabolomic profiling to identify potential biomarkers of metabolic syndrome

Juhan Pak[a,1], Mee-Hyun Lee[b,1], Seong-Eun Park[a], Soobin Bae[a], Gayoun Lee[a], Yanghee You[b], Gi Dae Kim[c], Chang-Su Na[b,*], and Hong-Seok Son[a,*]

[a] *Department of Biotechnology, Korea University, Seongbuk-gu, Seoul, 02841, South Korea*

[b] *College of Korean Medicine, Dongshin University, Naju, Jeollanam-do 58245, South Korea*

[c] *Department of Food and Nutrition, Kyungnam University, Changwon, Gyeongsangnam-do 51767, South Korea*

*Corresponding author:

Chang-Su Na (Tel: +82-16-330-3522, Email: csna@dsu.ac.kr)

Hong-Seok Son (Tel: +82-2-3290-3053, Email: sonhs@korea.ac.kr)

[1] These authors contributed equally to this work.

Supplementary Information:

Supplementary Tables

Table S1. Linear regression (R^2) and Spearman correlation between age and identified urinary metabolites. The *p*-value represents the significance of Spearman correlation.

No.	Metabolite	R^2	Spearman ρ	*p*_value
1	Galacturonic acid	0.0792	0.2679	0.0023
2	Sorbose	0.0498	0.2601	0.0031
3	Sulfuric acid	0.0485	0.2447	0.0056
4	Glucuronate	0.0650	0.2414	0.0063
5	Ribose	0.0434	0.2371	0.0073
6	Galactose	0.0366	0.2349	0.0079
7	Lyxose	0.0274	0.2235	0.0115
8	Cystine	0.0395	0.2110	0.0173
9	Lysine	0.0309	0.2095	0.0181
10	Xylonic acid	0.0442	0.2083	0.0188
11	Catechol	0.0374	0.2069	0.0196
12	Glycerol	0.0058	0.1872	0.0351
13	Inositol	0.0042	0.1851	0.0372
14	Glucose	0.0013	0.1818	0.0408
15	Xylose	0.0019	0.1811	0.0416
16	Fucose	0.0307	0.1757	0.0482
17	Tyrosine	0.0052	0.1739	0.0505
18	Citric acid	0.0235	0.1654	0.0631
19	Citramalic acid	0.0363	0.1653	0.0633
20	Pyruvate	0.0313	0.1646	0.0644
21	Indoxyl sulfate	0.0254	0.1619	0.0689
22	Phosphate	0.0133	-0.1591	0.0739
23	Maltose	0.0296	0.1589	0.0744
24	Galactitol	0.0069	0.1563	0.0793
25	Ascorbic acid	0.0014	0.1542	0.0834
26	Maltitol	0.0188	0.1484	0.0959
27	Daidzein	0.0075	0.1441	0.1061

28	Succinic acid	0.0125	0.1433	0.1080
29	Methylamine	0.0301	0.1415	0.1126
30	Threonic acid	0.0299	0.1389	0.1194
31	Hippurate	0.0085	0.1375	0.1233
32	Fumaric acid	0.0100	0.1351	0.1299
33	Lactose	0.0023	0.1341	0.1329
34	Threitol	0.0394	0.1295	0.1469
35	Aspartic acid	0.0035	0.1294	0.1471
36	Psicose	0.0117	0.1290	0.1484
37	Alanine	0.0331	0.1282	0.1508
38	Malic acid	0.0080	0.1279	0.1517
39	Ciliatine	0.0013	0.1254	0.1602
40	Mannitol	0.0000	0.1243	0.1640
41	Stearic acid	0.0158	0.1232	0.1676
42	3-Hydroxyphenylacetic acid	0.0023	0.1204	0.1776
43	Glyceric acid	0.0144	0.1197	0.1801
44	Palmitic acid	0.0124	0.1187	0.1838
45	Pyroglutamic acid	0.0213	0.1187	0.1838
46	Taurine	0.0053	0.1140	0.2019
47	2-Hydroxyisobutyric acid	0.0178	0.1026	0.2510
48	3-Hydroxybutyric acid	0.0111	0.1022	0.2528
49	Fructose	0.0121	0.1016	0.2557
50	Hippuric acid	0.0043	0.0948	0.2890
51	2-Hydroxybutyrate	0.0136	0.0947	0.2894
52	Valine	0.0141	0.0931	0.2978
53	Phenylalanine	0.0106	0.0907	0.3103
54	4-Hydroxyphenylacetic acid	0.0077	0.0890	0.3199
55	Gluconic acid	0.0093	0.0838	0.3490
56	Threonine	0.0184	0.0827	0.3555
57	Lactic acid	0.0009	0.0804	0.3690
58	3-Hydroxyisovaleric acid	0.0209	0.0767	0.3912
59	Uric acid	0.0056	0.0738	0.4096
60	Pantothenate	0.0128	0.0697	0.4360
61	Phenol	0.0077	0.0668	0.4555
62	Homovanillic acid	0.0021	0.0657	0.4629
63	Glucarate	0.0056	0.0628	0.4830

64	Propyleneglycol	0.0089	0.0625	0.4851
65	Glycine	0.0059	0.0622	0.4873
66	Tartaric acid	0.0005	0.0581	0.5164
67	Cadaverine	0.0081	0.0576	0.5202
68	2-Aminoethanol	0.0063	0.0573	0.5225
69	Shikimic acid	0.0205	0.0515	0.5651
70	Uracil	0.0002	-0.0487	0.5868
71	1-Methyl histidine	0.0007	0.0465	0.6034
72	Glycolic acid	0.0040	0.0445	0.6192
73	Pyrogallol	0.0052	-0.0390	0.6631
74	Acetaminophen glucuronide	0.0000	0.0259	0.7726
75	Histidine	0.0065	0.0254	0.7768
76	Hypoxanthine	0.0080	-0.0249	0.7810
77	1,6-Anhydroglucose	0.0015	0.0241	0.7880
78	3-Amino isobutyric acid	0.0000	-0.0184	0.8373
79	Creatinine	0.0027	0.0176	0.8446
80	Methyl succinic acid	0.0009	0.0136	0.8793

Table S2. The information of all identified metabolites in urine samples.

Peak No.	RT (min)	RI	Quant Mass	Metabolite	Standard Validation
1	4.011	939.21	171.07	Methylamine	
2	4.49	1001.80	117.09	Propyleneglycol	
3	5.081	1050.55	174.05	Pyruvate	
4	5.163	1057.25	151.05	Phenol	
5	5.234	1063.15	117.10	Lactic acid	O
6	5.293	1067.97	131.10	2-Hydroxyisobutyric acid	
7	5.403	1077.05	147.06	Glycolic acid	O
8	5.754	1106.28	116.10	Alanine	O
9	6.072	1133.46	147.07	2-Hydroxybutyrate	
10	6.435	1164.48	147.08	3-Hydroxybutyric acid	
11	6.493	1169.43	147.07	Sulfuric acid	
12	7.002	1213.94	131.10	3-Hydroxyisovaleric acid	
13	7.097	1222.69	144.12	Valine	
14	7.67	1275.33	174.10	2-Aminoethanol	
15	7.719	1279.80	147.08	Glycerol	
16	7.794	1286.72	299.07	Phosphate	
17	8.106	1316.66	174.10	Glycine	O
18	8.227	1328.64	254.10	Catechol	
19	8.233	1329.23	147.08	Succinic acid	O
20	8.253	1331.17	147.07	Methyl succinic acid	
21	8.33	1338.82	189.06	Glyceric acid	
22	8.411	1346.93	241.05	Uracil	
23	8.419	1347.65	245.05	Fumaric acid	O
24	8.911	1396.50	219.08	Threonine	O
25	9.629	1472.75	174.10	3-Aminoisobutyric acid	
26	9.747	1485.26	147.10	Citramalic acid	
27	9.842	1495.43	147.10	Malic acid	
28	10.047	1518.42	217.07	Threitol	
29	10.142	1529.17	232.10	Aspartic acid	
30	10.202	1535.96	156.10	Pyroglutamic acid	
31	10.425	1561.28	239.02	Pyrogallol	
32	10.437	1558.94	147.08	Threonic acid	
33	10.546	1575.02	115.10	Creatinine	
34	10.918	1618.16	164.07	3-Hydroxyphenylacetic acid	
35	11.13	1643.77	218.07	Phenylalanine	O

36	11.167	1648.07	252.11	4-Hydroxyphenylacetic acid	
37	11.212	1653.80	292.12	Tartaric acid	
38	11.399	1675.98	103.06	Xylose	O
39	11.452	1682.34	103.06	Lyxose	
40	11.514	1689.80	326.09	Taurine	
41	11.575	1697.26	103.06	Ribose	O
42	11.827	1729.65	217.08	1,6-Anhydroglucose	
43	11.836	1730.11	277.11	Indoxyl sulfate	
44	12.083	1761.48	398.14	Ciliatine	
45	12.129	1767.55	117.10	Fucose	
46	12.257	1783.64	326.10	Homovanillic acid	
47	12.42	1804.70	292.11	Xylonic acid	
48	12.511	1816.40	204.06	Shikimic acid	
49	12.548	1821.93	105.05	Hippurate	
50	12.561	1823.52	265.07	Hypoxanthine	
51	12.589	1827.49	96.10	1-Methyl histidine	
52	12.661	1836.87	273.08	Citric acid	
53	12.793	1854.57	105.05	Hippuric acid	
54	12.897	1868.29	174.11	Cadaverine	
55	13.017	1884.51	103.06	Psicose	
56	13.109	1896.80	103.06	Fructose	O
57	13.195	1908.49	103.06	Sorbose	
58	13.197	1908.93	319.15	Galactose	O
59	13.341	1929.18	319.14	Glucose	O
60	13.403	1938.08	156.13	Lysine	O
61	13.431	1942.02	154.10	Histidine	
62	13.545	1957.95	218.07	Tyrosine	
63	13.592	1964.52	319.13	Mannitol	O
64	13.648	1972.42	333.12	Glucuronate	
65	13.665	1974.92	332.10	Ascorbic acid	
66	13.668	1975.26	217.05	Galactitol	
67	13.779	1990.85	333.12	Galacturonic acid	
68	13.937	2013.61	103.06	Pantothenate	
69	14.119	2040.52	333.12	Gluconic acid	
70	14.16	2046.53	117.05	Palmitic acid	
71	14.236	2057.70	333.12	Glucarate	
72	14.682	2124.57	441.14	Uric acid	
73	14.724	2131.08	305.13	Inositol	
74	15.445	2244.21	117.05	Stearic acid	

75	15.922	2322.18	218.08	Cystine	O
76	18.337	2758.84	204.08	Lactose	
77	18.794	2849.07	204.08	Maltose	
78	18.85	2860.41	295.15	Acetaminophen glucuronide	
79	19.143	2917.99	361.16	Maltitol	
80	19.514	2986.82	398.10	Daidzein	

Table S3. Quantitative enrichment analysis (QEA) of MetS urine metabotype against metabolic pathway-associated metabolite sets.

Metabolite sets	Implicated metabolites [a]	Total [b]	Hits [c]	Raw *p* [d]	Holm *p* [e]	FDR [f]
Pentose and glucuronate interconversions	D-Xylose; D-Glucuronic acid	19	2	0.0040394	0.19389	0.16184
Fatty acid biosynthesis	Palmitic acid	47	1	0.015514	0.72917	0.16184
Fatty acid degradation	Palmitic acid	39	1	0.015514	0.72917	0.16184
Fatty acid elongation	Palmitic acid	38	1	0.015514	0.72917	0.16184
Biosynthesis of unsaturated fatty acids	Palmitic acid; Stearic acid	36	2	0.016859	0.74178	0.16184
Pentose phosphate pathway	D-Ribose; Gluconic acid; Glyceric acid	23	3	0.053654	1	0.42923
Inositol phosphate metabolism	Inositol; D-Glucuronic acid	30	2	0.086652	1	0.52014
Ascorbate and aldarate metabolism	Inositol; D-Glucuronic acid; Glucaric acid	9	3	0.08669	1	0.52014

Cysteine and methionine metabolism	L-Cystine; Pyruvic acid	33	2	0.12883	1	0.61006
Tyrosine metabolism	L-Tyrosine; Homovanillic acid; Pyruvic acid; Fumaric acid; p-Hydroxyphenylacetic acid;	42	5	0.15841	1	0.61006
Ubiquinone and other terpenoid-quinone biosynthesis	L-Tyrosine	18	1	0.16463	1	0.61006
Phenylalanine metabolism	Phenylalanine; L-Tyrosine	8	2	0.16523	1	0.61006
Phenylalanine, tyrosine and tryptophan biosynthesis	Phenylalanine; L-Tyrosine	4	2	0.16523	1	0.61006
Nicotinate and nicotinamide metabolism	L-Aspartic acid	15	1	0.26635	1	0.76805
Selenocompound metabolism	L-Alanine	20	1	0.29169	1	0.76805
Amino sugar and nucleotide sugar metabolism	D-Fructose; L-Fucose	42	2	0.30965	1	0.76805
Fructose and mannose metabolism	D-Fructose; L-Fucose	20	2	0.30965	1	0.76805

Starch and sucrose metabolism	D-Fructose; D-Glucose; D-Maltose	18	3	0.31645	1	0.76805
Neomycin, kanamycin and gentamicin biosynthesis	D-Glucose	2	1	0.31661	1	0.76805
Valine, leucine and isoleucine biosynthesis	L-Threonine; L-Valine	8	2	0.32621	1	0.76805
Pyruvate metabolism	Pyruvic acid; Malic acid; Fumaric acid	23	3	0.33602	1	0.76805
Glutathione metabolism	Glycine; Glutamic acid; Pyroglutamic acid; Cadaverine	28	4	0.40337	1	0.7905
Sulfur metabolism	Sulfate	8	1	0.40653	1	0.7905
Galactose metabolism	D-Galactose; Alpha-Lactose; D-Fructose; D-Glucose; Galactitol; Glycerol; Inositol	27	7	0.42713	1	0.7905
Glycine, serine and threonine metabolism	Glycine; L-Threonine; Glyceric acid; Pyruvic acid	33	4	0.44477	1	0.7905
Lipoic acid metabolism	Pyruvic acid; Glycine	28	2	0.44543	1	0.7905

Glycolysis / Gluconeogenesis	Pyruvic acid	26	1	0.45095	1	0.7905
Valine, leucine and isoleucine degradation	L-Valine	39	1	0.46113	1	0.7905
Porphyrin metabolism	Glycine; Glutamic acid	31	2	0.48768	1	0.80194
Pantothenate and CoA biosynthesis	Pantothenic acid; L-Valine; L-Aspartic acid; Uracil	20	4	0.50121	1	0.80194
Primary bile acid biosynthesis	Glycine; Taurine	46	2	0.5708	1	0.86804
Phosphonate and phosphinate metabolism	Ciliatine	6	1	0.5847	1	0.86804
Butanoate metabolism	3-Hydroxybutyric acid; Glutamic acid; Succinic acid	15	3	0.59678	1	0.86804
Arginine biosynthesis	Glutamic acid; L-Aspartic acid; Fumaric acid	14	3	0.71296	1	0.88292
Nitrogen metabolism	Glutamic acid	6	1	0.71316	1	0.88292

Arginine and proline metabolism	Glutamic acid; Pyruvic acid	36	2	0.71503	1	0.88292
Purine metabolism	Hypoxanthine; Sulfate; Uric acid	70	3	0.76213	1	0.88292
beta-Alanine metabolism	L-Aspartic acid; Uracil; Histidine	21	3	0.78759	1	0.88292
Biotin metabolism	Lysine	10	1	0.79331	1	0.88292
Lysine degradation	Lysine	30	1	0.79331	1	0.88292
Pyrimidine metabolism	Uracil	39	1	0.80336	1	0.88292
Citrate cycle (TCA cycle)	Succinic acid; Malic acid; Citric acid; Pyruvic acid; Fumaric acid	20	5	0.81855	1	0.88292
Histidine metabolism	Glutamic acid; Histidine; L-Aspartic acid	16	3	0.82877	1	0.88292
Glyoxylate and dicarboxylate metabolism	Citric acid; Malic acid; Glutamic acid; Glyceric acid; Pyruvic acid	31	6	0.83694	1	0.88292

Taurine and hypotaurine metabolism	Taurine	8	1	0.84117	1	0.88292
Glycerolipid metabolism	Glycerol; Glyceric acid	16	2	0.85992	1	0.88292
Alanine, aspartate and glutamate metabolism	L-Aspartic acid; L-Alanine; Glutamic acid; Citric acid; Fumaric acid; Pyruvic acid; Succinic acid	28	7	0.86453	1	0.88292
Propanoate metabolism	Succinic acid	21	1	0.9231	1	0.9231

[a] implicated metabolites from urine metabotype.

[b] total number of metabolites in the pathway.

[c] Hits: number of metabolites from MetS metabotype involved in the pathway.

[d] Raw *p*: original *p* value calculated from the pathway analysis.

[e] Holm *p*: adjusted raw *p* value by Holm-Bonferroni method.

[f] FDR: false discovery rate.

Table S4. Metabolic pathway associated with MetS urine metabotypes.

Metabolic pathway	Implicated metabolites [a]	Total [b]	Hits [c]	Raw *p* [d]	-Log (*p*) [e]	Holm *p* [f]	FDR [g]	Impact [h]
Pentose and glucuronate interconversions	D-Xylose; D-Glucuronate	19	2	0.004	2.394	0.194	0.194	0.17
Fatty acid biosynthesis	Palmitic acid	39	1	0.016	1.809	0.729	0.202	0.01
Fatty acid elongation	Palmitic acid	47	1	0.016	1.809	0.729	0.202	0.00
Biosynthesis of unsaturated fatty acids	Palmitic acid; Stearic acid	36	2	0.017	1.773	0.759	0.202	0.00
Pentose phosphate pathway	D-Ribose; D-Gluconic acid; D-Glycerate	23	3	0.054	1.270	1.000	0.515	0.04
Inositol phosphate metabolism	Inositol; D-Glucuronate	30	2	0.087	1.062	1.000	0.594	0.13
Ascorbate and aldarate metabolism	Inositol; D-Glucuronate; D-Glucarate	9	3	0.087	1.062	1.000	0.594	0.52
Cysteine and methionine metabolism	L-Cystine; Pyruvate	33	2	0.129	0.890	1.000	0.610	0.00

Fatty acid degradation	Palmitic acid; omega-Hydroxy fatty acid	39	2	0.134	0.873	1.000	0.610	0.00
Tyrosine metabolism	L-Tyrosine; Homovanillate; Pyruvate; Fumarate; 4-Hydroxyphenylacetate	42	5	0.158	0.800	1.000	0.610	0.17
Ubiquinone and other terpenoid-quinone biosynthesis	L-Tyrosine	18	1	0.165	0.783	1.000	0.610	0.00
Phenylalanine, tyrosine and tryptophan biosynthesis	L-Phenylalanine; L-Tyrosine	8	2	0.165	0.782	1.000	0.610	1.00
Phenylalanine metabolism	L-Phenylalanine; L-Tyrosine	4	2	0.165	0.782	1.000	0.610	0.36
Propanoate metabolism	Succinate; 2-Hydroxybutanoic acid	22	2	0.236	0.627	1.000	0.681	0.00
Nicotinate and nicotinamide metabolism	L-Aspartate	15	1	0.266	0.575	1.000	0.681	0.00
Pyruvate metabolism	Pyruvate; (S)-Malate; (S)-Lactate; Fumarate	23	4	0.287	0.543	1.000	0.681	0.22
Glycolysis / Gluconeogenesis	Pyruvate; (S)-Lactate	26	2	0.291	0.537	1.000	0.681	0.10

Selenocompound metabolism	L-Alanine	20	1	0.292	0.535	1.000	0.681	0.00
Fructose and mannose metabolism	D-Fructose; 6-Deoxy-L-galactose	42	2	0.310	0.509	1.000	0.681	0.10
Amino sugar and nucleotide sugar metabolism	6-Deoxy-L-galactose; D-Fructose	20	2	0.310	0.509	1.000	0.681	0.00
Starch and sucrose metabolism	D-Fructose; D-Glucose; Maltose	18	3	0.316	0.500	1.000	0.681	0.50
Neomycin, kanamycin and gentamicin biosynthesis	D-Glucose	2	1	0.317	0.499	1.000	0.681	0.00
Valine, leucine and isoleucine biosynthesis	L-Threonine; L-Valine	8	2	0.326	0.487	1.000	0.681	0.00
Glutathione metabolism	Glycine; L-Glutamate; pidolic acid; Cadaverine	28	4	0.403	0.394	1.000	0.763	0.12
Sulfur metabolism	Sulfate	8	1	0.407	0.391	1.000	0.763	0.21
Galactose metabolism	D-Galactose; Lactose; D-Fructose; D-Glucose; Galactitol; Glycerol; Inositol	27	7	0.427	0.369	1.000	0.763	0.50

Glycine, serine and threonine metabolism	Glycine; L-Threonine; D-Glycerate; Pyruvate	33	4	0.445	0.352	1.000	0.763	0.28
Lipoic acid metabolism	Pyruvate; Glycine	28	2	0.445	0.351	1.000	0.763	0.00
Valine, leucine and isoleucine degradation	L-Valine	40	1	0.461	0.336	1.000	0.763	0.00
Porphyrin metabolism	Glycine; L-Glutamate	31	2	0.488	0.312	1.000	0.776	0.00
Pantothenate and CoA biosynthesis	Pantothenate; L-Valine; L-Aspartate; Uracil	20	4	0.501	0.300	1.000	0.776	0.01
Primary bile acid biosynthesis	Glycine; Taurine	46	2	0.571	0.244	1.000	0.843	0.02
Phosphonate and phosphinate metabolism	2-Aminoethylphosphonate	6	1	0.585	0.233	1.000	0.843	0.00
Butanoate metabolism	(R)-3-Hydroxybutanoate; L-Glutamate; Succinate	15	3	0.597	0.224	1.000	0.843	0.00
Arginine biosynthesis	L-Glutamate; L-Aspartate; Fumarate	14	3	0.713	0.147	1.000	0.865	0.12

Nitrogen metabolism	L-Glutamate	6	1	0.713	0.147	1.000	0.865	0.00
Arginine and proline metabolism	L-Glutamate; Pyruvate	36	2	0.715	0.146	1.000	0.865	0.00
Purine metabolism	Hypoxanthine; Sulfate; Urate	70	3	0.762	0.118	1.000	0.865	0.02
beta-Alanine metabolism	L-Aspartate; Uracil; L-Histidine	21	3	0.788	0.104	1.000	0.865	0.00
Biotin metabolism	L-Lysine	10	1	0.793	0.101	1.000	0.865	0.00
Lysine degradation	L-Lysine	30	1	0.793	0.101	1.000	0.865	0.00
Pyrimidine metabolism	Uracil	39	1	0.803	0.095	1.000	0.865	0.05
Citrate cycle (TCA cycle)	Succinate; (S)-Malate; Citrate; Pyruvate; Fumarate	20	5	0.819	0.087	1.000	0.865	0.24
Glyoxylate and dicarboxylate metabolism	Citrate; (S)-Malate; Glycine; L-Glutamate; D-Glycerate; Pyruvate	32	6	0.837	0.077	1.000	0.865	0.22

Taurine and hypotaurine metabolism	Taurine	8	1	0.841	0.075	1.000	0.865	0.43
Histidine metabolism	L-Glutamate; L-Histidine; N(pi)-Methyl-L-histidine; L-Aspartate	16	4	0.843	0.074	1.000	0.865	0.22
Glycerolipid metabolism	Glycerol; D-Glycerate	16	2	0.860	0.066	1.000	0.865	0.33
Alanine, aspartate and glutamate metabolism	L-Aspartate; L-Alanine; Citrate; Fumarate; Pyruvate; Succinate	28	7	0.865	0.063	1.000	0.865	0.42

[a] Implicated metabolites from urine metabotype.

[b] Total number of metabolites in the pathway.

[c] Hits: number of metabolites from MetS metabotype involved in the pathway.

[d] Raw *p*: original *p* value calculated from the pathway analysis.

[e] -log(*p*): negative log of *p* value.

[f] Holm *p*: adjusted raw *p* value by Holm-Bonferroni method.

[g] FDR: false discovery rate.

[h] Impact: impact of the pathway as calculated from pathway topology analysis.

Table S5. Confusion matrix-based performance metrics of urinary metabolite-derived MetS prediction model stratified by diagnostic criteria.

Criterion	Level	n[a]	TP[b]	FP[c]	FN[d]	TN[e]	Accuracy[f]	Sensitivity[g]	Specificity[h]	Precision[i]	F1[j]
WC	normal	72	2	24	2	44	0.64	0.65	0.50	0.96	0.77
	abnormal	53	21	0	30	2	0.43	1.00	0.41	0.06	0.12
BP	normal	58	2	18	3	35	0.64	0.66	0.40	0.92	0.77
	abnormal	69	21	6	29	13	0.49	0.68	0.42	0.31	0.43
HDL	normal	70	1	22	2	45	0.66	0.67	0.33	0.96	0.79
	abnormal	17	4	2	8	3	0.41	0.60	0.33	0.27	0.38
TG	normal	72	3	24	4	41	0.61	0.63	0.43	0.91	0.75
	abnormal	15	2	0	6	7	0.60	1.00	0.25	0.54	0.70
BG	normal	55	1	16	5	33	0.62	0.67	0.17	0.87	0.76
	abnormal	72	22	8	27	15	0.51	0.65	0.45	0.36	0.46

[a] n: Number of participants in each subgroup (normal or abnormal).

[b] TP (True Positive): MetS cases correctly predicted as MetS.

[c] FP (False Positive): Non-MetS cases incorrectly predicted as MetS.

[d] FN (False Negative): MetS cases incorrectly predicted as Non-MetS.

[e] TN (True Negaive): Non-MetS cases correctly predicted as Non-MetS

[f] Accuracy: Proportion of correctly classified cases among all cases.

[g] Sensitivity: Proportion of true MetS cases correctly predicted as MetS.

[h] Specificity: Proportion of non-MetS cases correctly predicted as non-MetS.

[i] Precision: Proportion of predicted MetS cases that were true MetS.

[j] F1 score: Harmonic mean of Precision and Sensitivity.

Supplementary figures

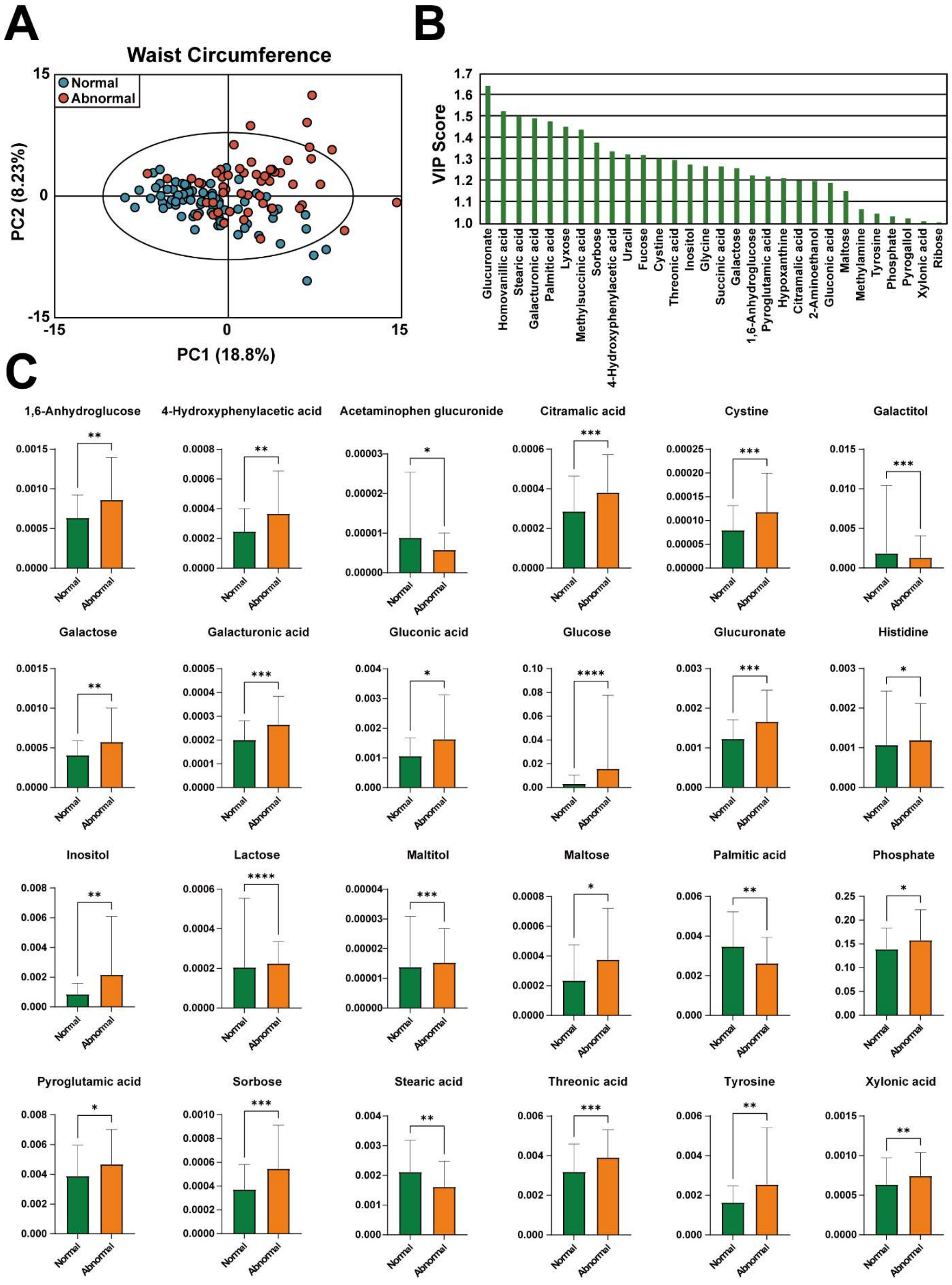

Fig. S1. Multivariate analysis and difference in metabolite levels between normal and abnormal groups based on waist circumference. (A) PLS-DA plot represents the separation of participants based on their metabolic profiles in relation to waist circumference. (B) VIP score plot represents the metabolites contributing to group separation (VIP ≥ 1.0). (C) Bar plots showing the significantly different metabolites between the two groups (*: $p < 0.05$).

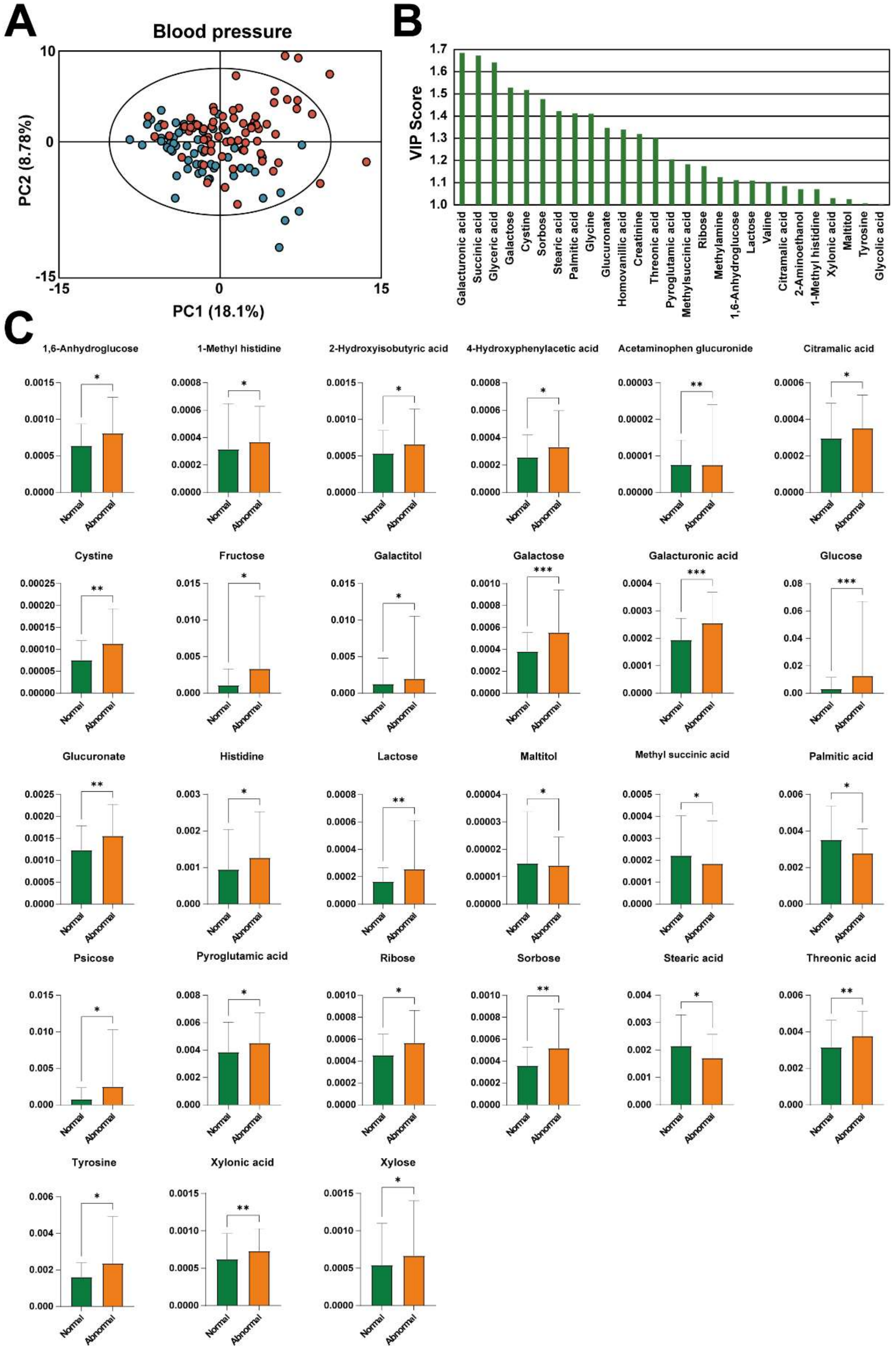

A
Blood pressure
PC1 (18.1%)
PC2 (8.78%)
B
VIP Score
Galacturonic acid
Succinic acid
Glyceric acid
Galactose
Cystine
Sorbose
Stearic acid
Palmitic acid
Glycine
Glucuronate
Homovanillic acid
Creatinine
Threonic acid
Pyroglutamic acid
Methylsuccinic acid
Ribose
Methylamine
1,6-Anhydroglucose
Lactose
Valine
Citramalic acid
2-Aminoethanol
1-Methyl histidine
Xylonic acid
Maltitol
Tyrosine
Glycolic acid
C
1,6-Anhydroglucose
1-Methyl histidine
2-Hydroxyisobutyric acid
4-Hydroxyphenylacetic acid
Acetaminophen glucuronide
Citramalic acid
Cystine
Fructose
Galactitol
Galactose
Galacturonic acid
Glucose
Glucuronate
Histidine
Lactose
Maltitol
Methyl succinic acid
Palmitic acid
Psicose
Pyroglutamic acid
Ribose
Sorbose
Stearic acid
Threonic acid
Tyrosine
Xylonic acid
Xylose
Normal
Abnormal

Fig. S2. Multivariate analysis and difference in metabolite levels between normal and abnormal groups based on blood pressure. (A) PLS-DA plot represents the separation of participants based on their metabolic profiles in relation to blood pressure. (B) VIP score plot represents the metabolites contributing to group separation (VIP ≥ 1.0). (C) Bar plots showing the significantly different metabolites between the two groups (*: $p < 0.05$, **: $p < 0.01$).

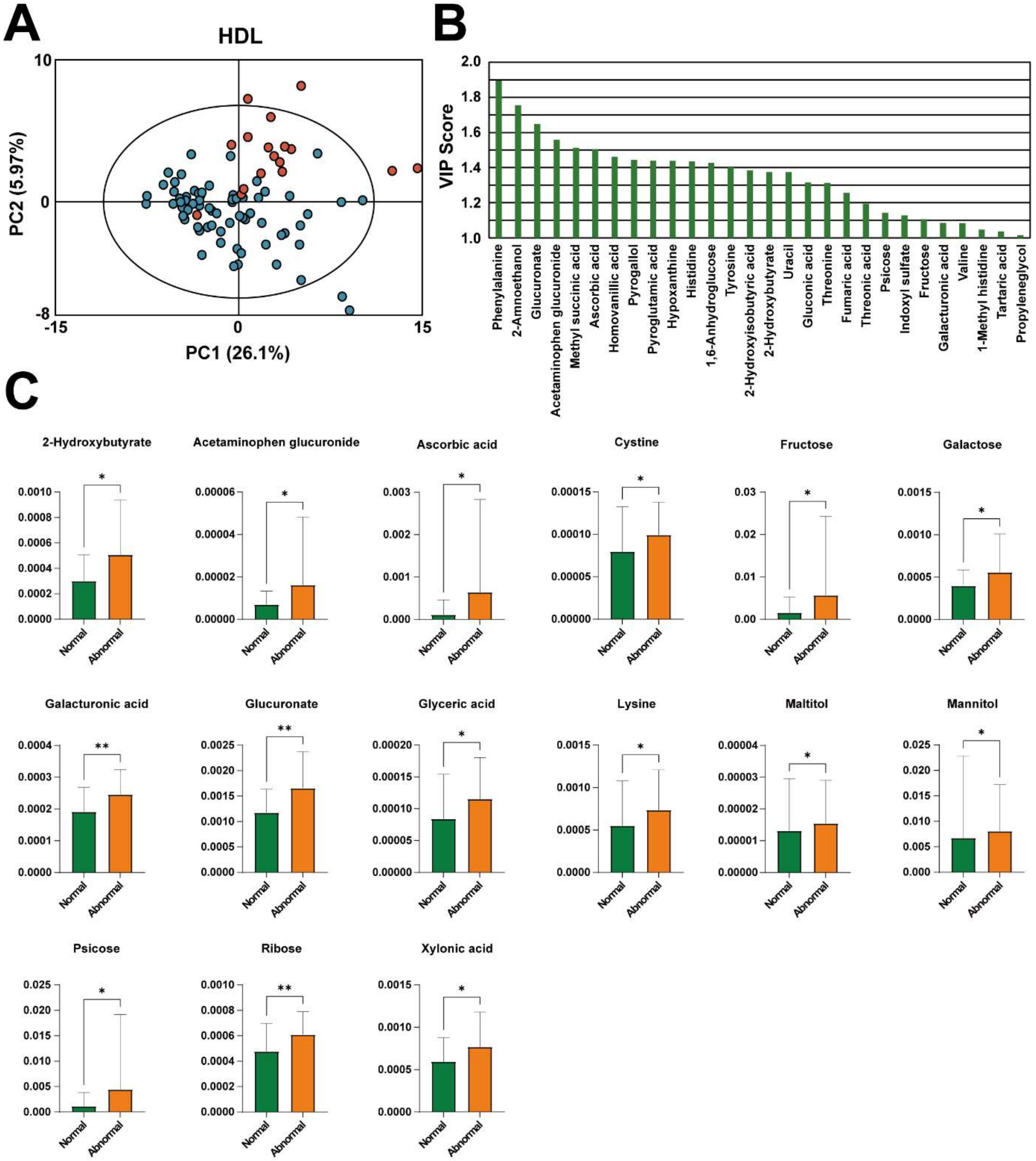

Fig. S3. Multivariate analysis and difference in metabolite levels between normal and abnormal groups based on HDL. (A) PLS-DA plot represents the separation of participants based on their metabolic profiles in relation to HDL. (B) VIP score plot represents the metabolites contributing to group separation (VIP ≥ 1.0). (C) Bar plots showing the significantly different metabolites between the two groups (*: $p < 0.05$, **: $p < 0.01$).

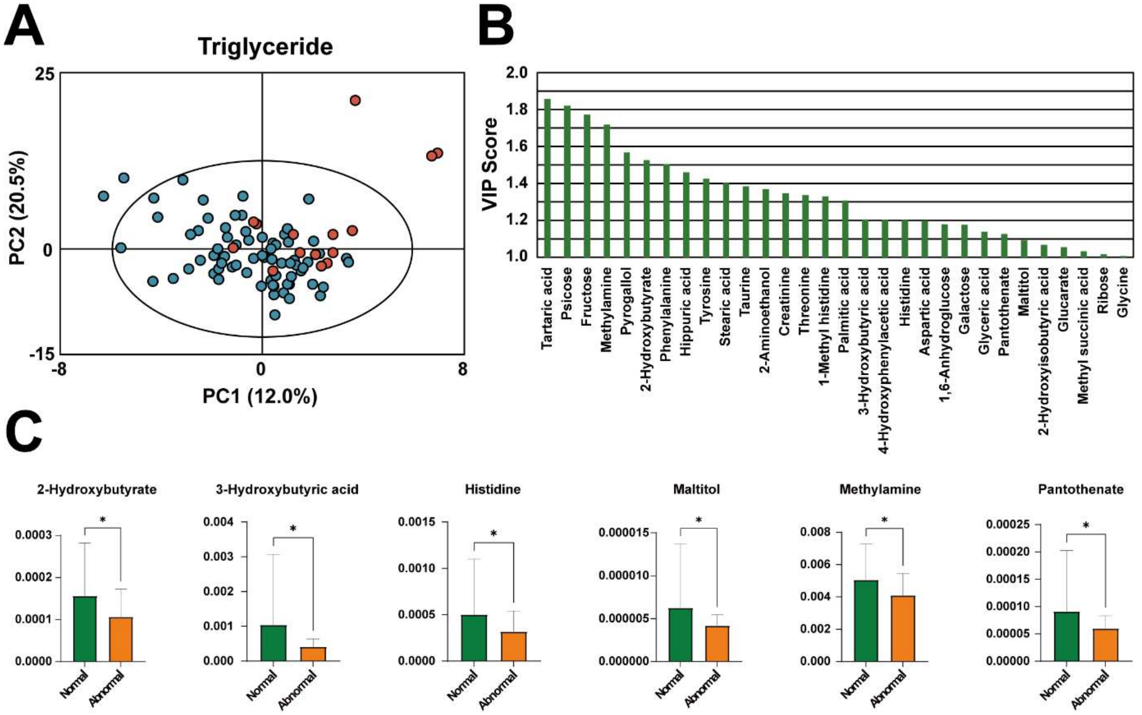

Fig. S4. Multivariate analysis and difference in metabolite levels between normal and abnormal groups based on triglyceride. (A) PLS-DA plot represents the separation of participants based on their metabolic profiles in relation to triglyceride. (B) VIP score plot represents the metabolites contributing to group separation (VIP ≥ 1.0). (C) Bar plots showing the significantly different metabolites between the two groups (*: $p < 0.05$, **: $p < 0.01$).

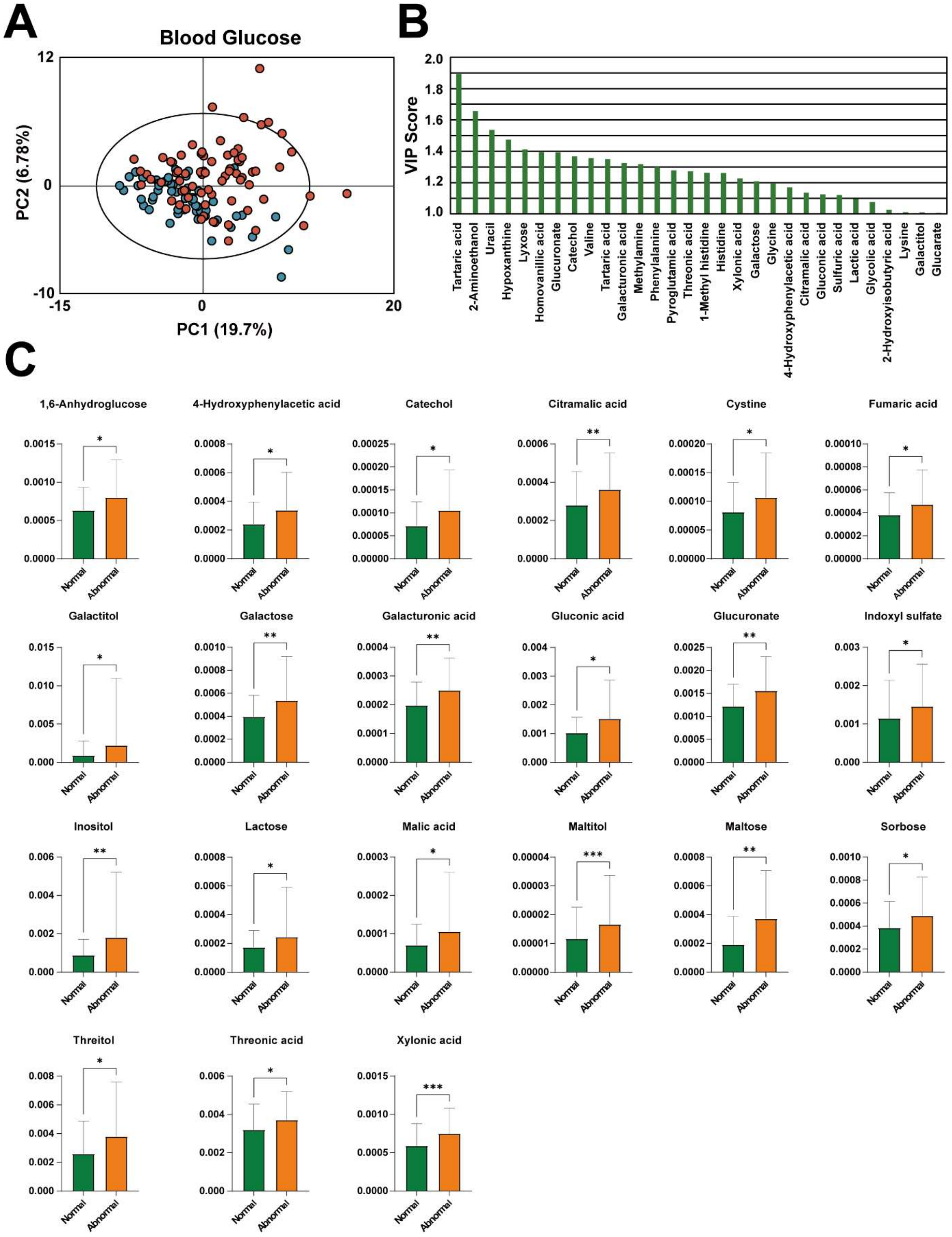

Fig. S5. Multivariate analysis and difference in metabolite levels between normal and abnormal groups based on fasting blood glucose. (A) PLS-DA plot represents the separation

of participants based on their metabolic profiles in relation to fasting blood glucose. (B) VIP score plot represents the metabolites contributing to group separation (VIP ≥ 1.0). (C) Bar plots showing the significantly different metabolites between the two groups (*: $p < 0.05$, **: $p < 0.01$).

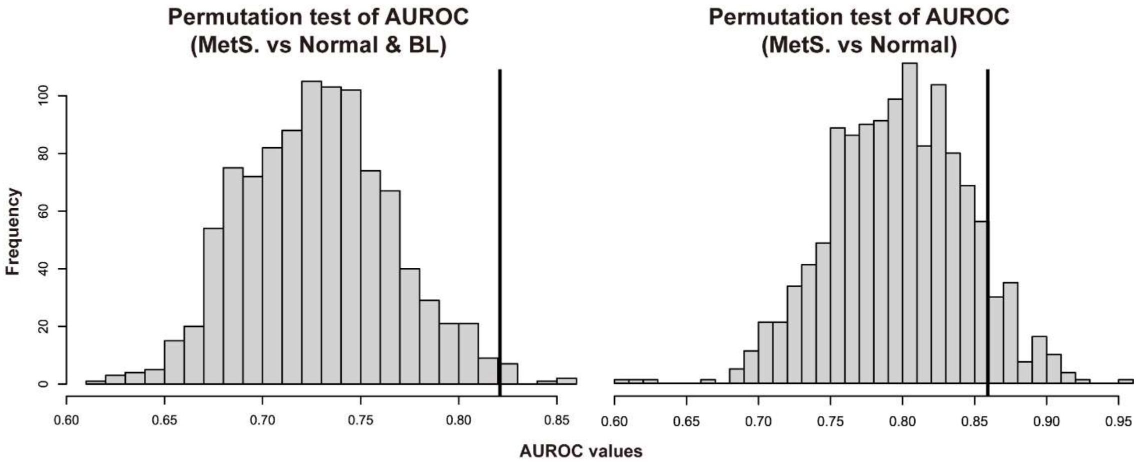

Fig. S6. Permutation tests validating AUROC performance of multivariable prediction models. (A) Null distribution of AUROC values obtained from 1,000 label permutation for the multivariable logistic regression model distinguishing MetS from the combined non-MetS group (Normal & BL). The observed AUROC (0.82) is indicated by a vertical line and was significantly higher than expected under the null distribution (p < 0.001). (B) Null distribution of AUROC values obtained from 1,000 label permutations for the model distinguishing MetS from the Normal group only. The observed AUROC (0.86) is indicated by a vertical line and was also significantly higher than expected under the null distribution (p < 0.001).
